# Supplementary material for: Methods for high-dimensonal analysis of cells dissociated from cyropreserved synovial tissue
Source: Arthritis Res Ther. 2018 Jul 11;20:139. doi: 10.1186/s13075-018-1631-y (PMC6042350; doi:10.1186/s13075-018-1631-y)
Supplement: Supplementary file 1 — Table S1. Mass cytometry panel for analysis of synovial cells. Figure S1. Additional variables in synovial tissue enzymatic treatment (PDF 2239 kb) [file 13075_2018_1631_MOESM1_ESM.pdf]

Metal	Target	Clone
141Pr	CD45	HI30
142Nd	CD19	HIB19
143Nd	RANKL	MIH24
144Nd	CD64	10.1
145Nd	CD16	3G8
146Nd	CD8α	RPA T8
147Sm	FAP	Poly
148Nd	CD20	2H7
149Sm	CD45RO	UCHL1
150Nd	CD38	HIT2
151Eu	PD-1	EH12.2H7
152Sm	CD14	M5E2
153Eu	CD69	FN50
154Sm	CXCR5	J252D4
155Gd	CD4	RPA T4
156Gd	Podoplanin	NC-08
158Gd	CD3	UCHT1
159Tb	CD11c	Bu15
160Gd	FcRL4	413D12
161Dy	CD138	MI15
162Dy	CD90	5E 10
163Dy	CCR2	K036C2
164Dy	Cadherin11	23C6
165Ho	FoxP3	PCH101
166Er	CD34	581
167Er	CD146	SHM-57
168Er	IgA	9H9H11
169Tm	TCRgd	B1
170Er	ICOS	C398.4A
171Yb	CD66b	G10F5
172Yb	IgM	MHM-88
173Yb	CD144	BV9
174Yb	HLA-DR	L243
175Lu	IgD	IA6-2
176Yb	VCAM-1	STA
195Pt	Live/Dead	Cell-ID

Table S1. Mass cytometry panel for analysis of synovial cells.

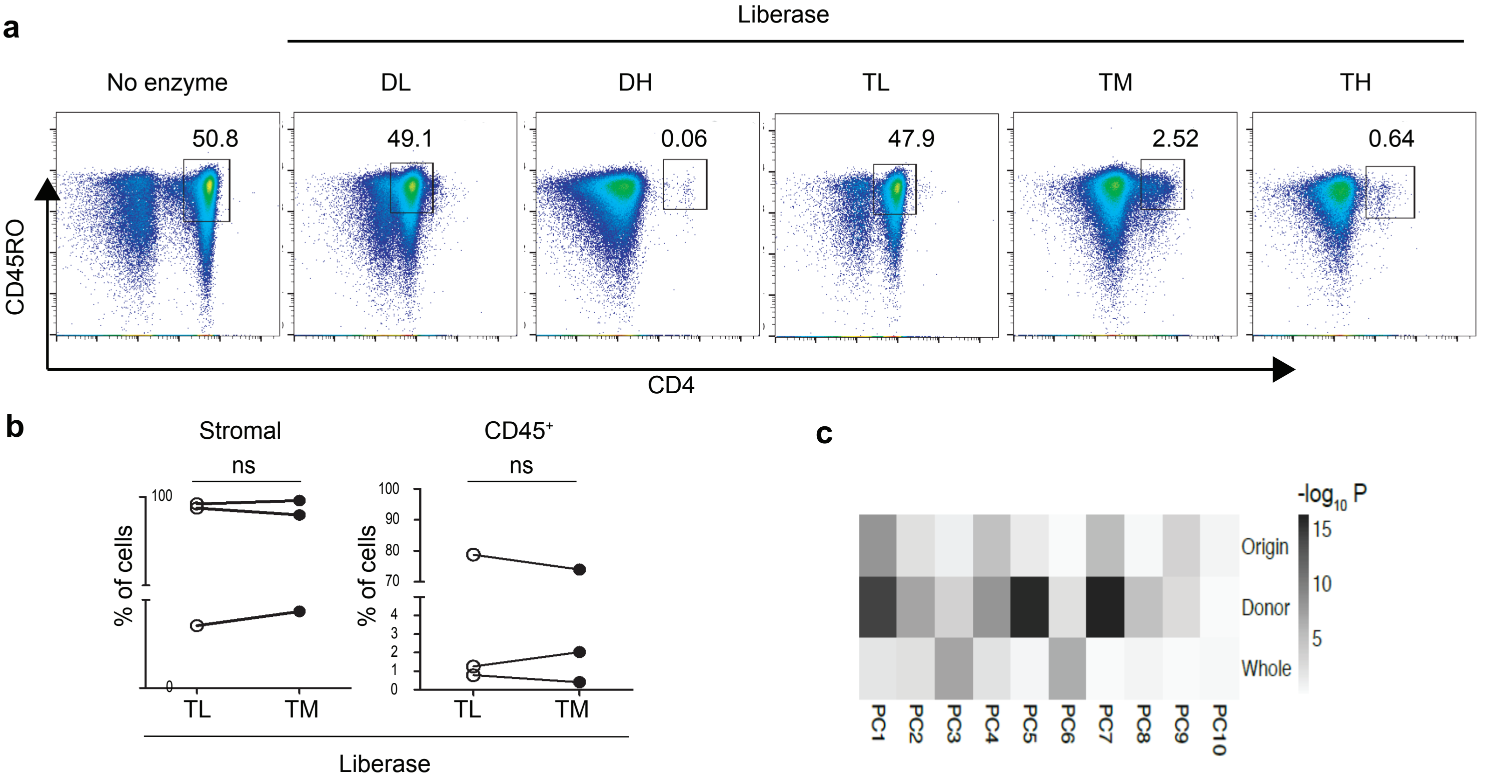

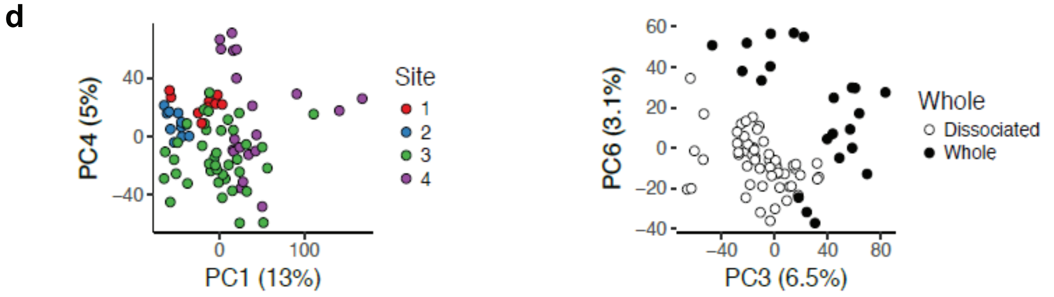

e

Gene	Fold	Adj p-value
DNAJB1	2.1	4.78E-27
ATF3	3.1	8.07E-25
JUN	2.3	9.31E-24
HSPA6	2.3	3.56E-23
HSPA1B	2.3	3.56E-23
FOSB	2.5	3.67E-18
HSPA1A	1.9	5.54E-18
FOS	2	7.16E-12
ZFP36	1.4	1.04E-09
EGR1	1.8	1.36E-08

Figure S1. Additional variables in synovial tissue enzymatic treatment.
a) Flow cytometry analysis of human peripheral blood mononuclear cells after incubation with Liberase proteolytic enzyme formulations. Cells were first gated for viability and CD3 T cell receptor expression. b) Synovial tissue collected during arthroplasty surgery was mechanically disrupted and treated with or without a panel of Liberase proteolytic enzymes. Dissociated cells were analyzed by flow cytometry. Representative data from four biological replicates. ns, not significantly different. c) Principal component scores for variables in synovial tissue processing bulk (nonsorted) synoviocyte RNA-seq transcriptomics. d) Principal component analysis on disaggregated synoviocyte and whole tissue transcriptomics, color coded based on the clinical collection site for each sample (left panel) or whether the sequencing was from dissociated or whole/intact tissue. e) Stress response genes expressed higher in dissagregated synovial samples compared to whole tissue.The heatmap plots the –log10 P values associated with each factor for each principal component.
